# Supplementary material for: Computational Modelling of Cancer Nanomedicine: Integrating Hyperthermia Treatment Into a Multiphase Porous‐Media Tumour Model
Source: Int J Numer Method Biomed Eng. 2025 Aug 5;41(8):e70074. doi: 10.1002/cnm.70074 (PMC12325832; doi:10.1002/cnm.70074)
Supplement: Supplementary file 1 — Data S1. Supporting Information. [file CNM-41-e70074-s001.pdf]

Supporting Information

Computational Modelling of Cancer Nanomedicine: Integrating Hyperthermia Treatment Into a Multiphase Porous-Media Tumour Model

Barbara Wirthl | Paolo Decuzzi | Bernhard A. Schrefler | Wolfgang A. Wall

Boundary Conditions of the Numerical Examples

Example 3.1 | Idealised Spherical Tumour With Lumped Heat Sink Term

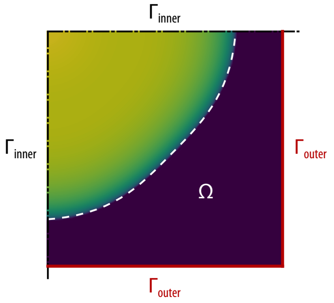

Boundary conditions		
Nanoparticle transport		
Dirichlet BC on Ω	ω_D^{NPv}	$1.0 \cdot 10^{-3}$, $1.5 \cdot 10^{-3}$, $2.0 \cdot 10^{-3}$
Temperature		
Neumann BC on Γ_{inner}	$\kappa_{eff}\, \boldsymbol{\nabla} T \cdot \boldsymbol{n} = 0$	
Robin BC on Γ_{outer}	$-\kappa_{eff}\, \boldsymbol{\nabla} T \cdot \boldsymbol{n} = h\,(T - T_b)$	$h = 2.0 \cdot 10^{-5}$ W/(mm^{2}K)<br>$T_b = 37$ °C

Example 3.2 | Tumour With a Discrete Microvascular Network

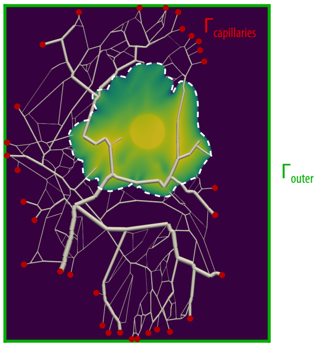

Boundary conditions		
Nanoparticle transport		
Dirichlet BC on $\Gamma_{capillaries}$	$\omega_D^{NP\hat{v}}$	$2.0 \cdot 10^{-3}$
Temperature		
Robin BC on Γ_{outer}	$-\kappa_{eff}\, \boldsymbol{\nabla} T \cdot \boldsymbol{n} = h\,(T - T_b)$	h see Section 3.2<br>$T_b = 37$ °C

Example 3.3 | *In vivo* Tumour in a Mouse Model

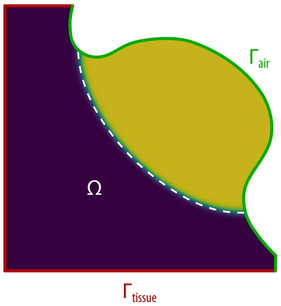

Boundary conditions		
Nanoparticle transport		
Dirichlet BC on Ω	ω_D^{NPv}	see Section 3.3
Temperature		
Robin BC on Γ_{tissue}	$-\kappa_{eff}\, \boldsymbol{\nabla} T \cdot \boldsymbol{n} = h\,(T - T_b)$	$h = 2.0 \cdot 10^{-5}$ W/(mm^{2}K), $T_b = 29$ °C
Robin BC on Γ_{air}	$-\kappa_{eff}\, \boldsymbol{\nabla} T \cdot \boldsymbol{n} = h\,(T - T_b)$	$h = 0.3 \cdot 10^{-5}$ W/(mm^{2}K), $T_b = 29$ °C
